# Impact of SARS-Cov-2 exposure during pregnancy on child neurodevelopment: systematic review and meta-analysis

**DOI:** 10.1590/1984-0462/2025/43/2025094

**Published:** 2025-12-12

**Authors:** Paloma Alves Miquilussi, Thalis Vasconcellos de Aguiar, Paula Lima da Cruz, Ana Paula Duca, Marcos Ribeiro, Cristiano Miranda de Araújo, Rosane Sampaio Santos, Angela Graciela Deliga Schroder, Ana Chrystina de Souza Crippa

**Affiliations:** a Universidade Tuiuti do Paraná, Curitiba, PR, Brazil.; b Universidade Federal do Paraná, Curitiba, PR, Brazil.

**Keywords:** COVID-19, Neurodevelopmental disorders, Pregnancy complications, Infectious, COVID-19, Transtornos do neurodesenvolvimento, Complicações na Gravidez, Infecciosa

## Abstract

**Objective:**

The objective of this study was to investigate the impact of intrauterine exposure to SARS-CoV-2 on neurodevelopment in children up to 2 years of age.

**Data source:**

A combination of keywords and truncations was adapted for five electronic databases: Excerpta Medica Database (EMBASE), Latin American and Caribbean Literature in Health Sciences (LILACS), PubMed/Medline, Scopus, and Web of Science. Additionally, gray literature sources were consulted, including the American Speech-Language-Hearing Association (ASHA), Google Scholar, and ProQuest Dissertations and Theses. The quality of evidence was assessed using the Joanna Briggs Institute's Critical Appraisal Checklist. A random-effects meta-analysis was performed to evaluate the primary outcome using R in R Studio version 1.2.1335 (RStudio Inc., Boston, USA). Study weights were calculated using the Mantel-Haenszel method, and variance, expressed by Tau^2^ values, employed the DerSimonian-Laird estimator. The Freeman-Tukey double arcsine transformation was applied to approximate a normal data distribution, and 95% confidence intervals were calculated for each meta-analysis.

**Data synthesis:**

Nine articles were included in the qualitative synthesis and eight in the quantitative analysis. There was no statistically significant difference in neurodevelopment between infants exposed and unexposed to SARS-CoV-2 during pregnancy [RR=5.10; 95%CI 0.36–71.85; I^2^=91%]. However, for the "fine motor" domain, the exposed group had a 2.38 times higher risk of deficit [95%CI 1.22–4.68] compared to the non-exposed group, with low heterogeneity in the analysis (I^2^=0%).

**Conclusions:**

Exposure to the SARS-CoV-2 virus during the gestational period is not associated with neurodevelopmental delay up to 2 years of age, although it has been linked to an increased risk of delayed fine motor development. However, this evidence remains uncertain due to the limited number of studies on the topic and the heterogeneity of methodologies.

## RESUMO

**Objetivo:**

Investigar o impacto da exposição intrauterina ao SARS-CoV-2 no neurodesenvolvimento de crianças até os dois anos de idade.

**Fontes de dados:**

Foram utilizadas combinações de palavras-chave e truncamentos em cinco bases de dados: Excerpta Medica database (EMBASE), Latin American and Caribbean Literature in Health Sciences (LILACS), United States National Library of Medicine/Medical Literature Analysis and Retrieval System Online (PubMed/Medline), Scopus e Web of Science. Além disso, literaturas cinzentas foram consultadas, incluindo por meio da American Speech-Language-Hearing Association (ASHA), Google Scholar e Proquest Dissertation and Thesis. A qualidade da evidência foi avaliada com o uso da Joanna Briggs Institute's Critical Appraisal Checklist. Uma metanálise de efeito aleatório foi conduzida utilizando a linguagem R no R Studio version 1.2.1335 (Rstudio Inc, Boston, USA). O peso dos estudos foi calculado pelo método Mantel-Haenszel, enquanto a variância (Tau^2^) foi estimada por DerSimonian-Laird. Para normalizar os dados, aplicou-se o método do arcoseno duplo de Freeman-Tukey. Intervalos de confiança de 95% foram calculados para cada metanálise.

**Síntese dos dados:**

Foram incluídos nove artigos na síntese qualitativa e oito na quantitativa. Não houve diferença estatisticamente significativa no neurodesenvolvimento entre bebês expostos e não expostos ao SARS-CoV-2 na gestação [risco relativo — RR=5,10; intervalo de confiança de 95% — IC95% 0,36–71,85; I^2^=91%]. No entanto, para o domínio "motricidade fina", o grupo exposto apresentou risco 2,38 vezes maior de déficit [IC95% 1,22–4,68] em comparação ao grupo não exposto, com baixa heterogeneidade na análise (I^2^=0%).

**Conclusões:**

A exposição ao SARS-CoV-2 na gestação não se mostrou associada a atraso no neurodesenvolvimento, embora tenha sido relacionada a maior risco de atraso no desenvolvimento da motricidade fina. Contudo, essa evidência ainda é incerta em razão da limitação de estudos e da heterogeneidade metodológica.

## INTRODUCTION

The COVID-19 pandemic, caused by the SARS-CoV-2 virus, has brought significant consequences to global public health, particularly impacting vulnerable groups such as pregnant women and their babies.^1-3^ The uncertainty regarding the risks of this infection has raised concerns about potential adverse effects, both immediate and long term, on maternal health and child neurodevelopment.^4-6^

The mechanisms by which SARS-CoV-2 may influence the neurodevelopment of children exposed during pregnancy appear to be more closely related to the activation of the maternal immune system than to the vertical transmission of the virus.^7^ Although vertical transmission is rare and evidence suggests that breastfeeding does not pose a transmission risk, the indirect effects of the infection remain a cause for concern.^8-10^ Previous studies indicate that viral infections and stress during pregnancy are associated with adverse clinical outcomes for mothers and children, including changes in child neurodevelopment.^11-14^ Furthermore, historical reports of previous pandemics show that children born in these contexts may exhibit neuropsychological sequelae that often only become evident after years of follow-up.^7^

In the context of COVID-19, preliminary evidence suggests an association between infection during pregnancy and obstetric complications, such as preterm birth, preeclampsia, intrauterine growth restriction, and the need for neonatal intensive care.^15,16^ These conditions have previously been linked to negative impacts on child neurodevelopment. However, the available data on the direct and indirect effects of SARS-CoV-2 infection on child development remain limited and, in some cases, they are contradictory.^17,18^ Although some studies have not identified significant differences in infant neurodevelopmental scores,^4,5^ others suggest that early exposure to the virus may increase the risks of neurodevelopmental changes, particularly when the infection occurs in the early stages of pregnancy.^19^

Even modest increases in the risks of neurodevelopmental changes can have significant impacts on public health.^7^ reinforcing the need for monitoring programs aimed at children exposed to COVID-19 in utero. Considering this, the aim of this systematic review was to investigate the impact of COVID-19 on the neurodevelopment of children exposed to SARS-CoV-2 during pregnancy, addressing the following question: What is the impact of COVID-19 on the neurodevelopment of children exposed in utero?

## METHOD

This systematic review was developed in accordance with the Preferred Reporting Items for Systematic Reviews and Meta-Analyses (PRISMA) statement.^20^

To determine the eligibility of the studies included in this review, the "PECOS" acronym was used, with the following criteria: Population (P) – Children up to 2 years of age; Exposure (E) – Exposed to SARS-CoV-2 during pregnancy; Comparison (C) – Not exposed to SARS-CoV-2 or studies without a comparator; Outcome (O) – Neurodevelopmental disorders; Study design (S) – Observational studies.

Studies that assessed the neurodevelopment of babies (0–2 years) born to mothers infected with SARS-CoV-2 during any period of pregnancy were included. Observational studies with a control group of uninfected mothers were included, as well as prevalence studies without a control group. There were no restrictions regarding language, country, or publication period.

Studies that analyzed children with pre-existing comorbidities involving the central nervous system, associated with underlying diseases other than COVID-19, or those with peripartum complications unrelated to SARS-CoV-2 infection were excluded. Studies that did not provide a direct assessment of the neurodevelopment of children exposed to SARS-CoV-2, or that did not use the ASQ-3, or Bayley III scales in their analyses were also excluded. Additionally, studies with incomplete or inconsistent data, as well as publications in non-primary formats such as conference proceedings, case reports, case series, narrative reviews, letters to the editor, and expert opinions, were discarded.

The combination of keywords and truncations was adapted for the five selected electronic databases as sources of information: Excerpta Medica Database (EMBASE), Latin American and Caribbean Literature in Health Sciences (LILACS), PubMed/Medline, Scopus, and Web of Science. Additionally, gray literature was consulted, including the American Speech-Language-Hearing Association (ASHA), Google Scholar, and ProQuest Dissertation and Thesis (Table 1). The database searches were conducted on December 4, 2023, and the grey literature search on January 18, 2024. Additionally, an updated search was conducted in January 2025. No date limits were applied. The reference lists of the articles included were also reviewed through Citation Chaser.^21^ An expert in the field was consulted to suggest any relevant publications for this study, which were then assessed for eligibility. A reference manager (EndNote^®^ X7, Thomson Reuters, Philadelphia, PA) was used to remove duplicate references.

**Table 1:** Database search strategy.

Database	Search (October 2023) Updated
EMBASE	("COVID-19" OR "SARS-CoV-2" OR "Severe Acute Respiratory Syndrome Coronavirus 2" OR "NCOV" OR "2019 NCOV" OR "severe acute respiratory syndrome" OR "severe acute respiratory syndrome-related coronavirus") AND ("Neurodevelopment" OR "neurodevelopmental disorders" OR "developmental disabilities" OR "developmental disorder")
LILACS	("COVID-19" OR "SARS-CoV-2" OR "Severe Acute Respiratory Syndrome Coronavirus 2"OR "NCOV" OR "2019 NCOV" OR "severe acute respiratory syndrome" OR "severe acute respiratory syndrome-related coronavirus" OR "Síndrome Respiratória Aguda Grave Coronavírus 2" OR "Síndrome Respiratorio Agudo Severo Coronavirus 2" OR "síndrome respiratória aguda grave" OR "Síndrome respiratorio agudo severo" OR "coronavírus relacionado à síndrome respiratória aguda grave" OR "coronavirus relacionado con el síndrome respiratorio agudo severo") AND ("Neurodevelopment" OR "neurodevelopmental disorders" OR "developmental disabilities" OR "developmental disorder" OR "Neurodesenvolvimento" OR "Neurodesarrollo" OR "distúrbios do neurodesenvolvimento" OR "trastornos del neurodesarrollo" OR "deficiências de desenvolvimento" OR "discapacidades del desarrollo" OR "transtorno de desenvolvimento" OR "trastorno del desarrollo")
PubMed	("COVID-19"[All Fields] OR "COVID-19"[MeSH Terms] OR "SARS-CoV-2"[All Fields] OR "sars-cov-2"[MeSH Terms] OR "Severe Acute Respiratory Syndrome Coronavirus 2"[All Fields] OR "NCOV"[All Fields] OR "2019 NCOV"[All Fields] OR "severe acute respiratory syndrome"[MeSH Terms] OR "Severe Acute Respiratory Syndrome"[Text Word] OR "severe acute respiratory syndrome-related coronavirus"[MeSH Terms] OR "Severe acute respiratory syndrome-related coronavirus"[Text Word]) AND ("Neurodevelopment"[All Fields] OR "neurodevelopmental"[All Fields] OR "neurodevelopmental disorders"[MeSH Terms] OR "Neurodevelopmental Disorders"[Text Word] OR "developmental disabilities"[MeSH Terms] OR "developmental disorder"[Text Word])
Scopus	TITLE-ABS-KEY ("COVID-19" OR "SARS-CoV-2" OR "Severe Acute Respiratory Syndrome Coronavirus 2" OR "NCOV" OR "2019 NCOV" OR "severe acute respiratory syndrome" OR "severe acute respiratory syndrome-related coronavirus") AND TITLE-ABS-KEY("Neurodevelopment" OR "neurodevelopmental disorders" OR "developmental disabilities" OR "developmental disorder")
Web of Science	TS=("COVID-19" OR "SARS-CoV-2" OR "Severe Acute Respiratory Syndrome Coronavirus 2" OR "NCOV" OR "2019 NCOV" OR "severe acute respiratory syndrome" OR "severe acute respiratory syndrome-related coronavirus") AND TS=("Neurodevelopment" OR "neurodevelopmental disorders" OR "developmental disabilities" OR "developmental disorder")
LIVIVO	("COVID-19" OR "SARS-CoV-2" OR "Severe Acute Respiratory Syndrome Coronavirus 2" OR "NCOV" OR "2019 NCOV" OR "severe acute respiratory syndrome" OR "severe acute respiratory syndrome-related coronavirus") AND ("Neurodevelopment" OR "neurodevelopmental disorders" OR "developmental disabilities" OR "developmental disorder")
ProQuest	NOFT("COVID-19" OR "SARS-CoV-2" OR "Severe Acute Respiratory Syndrome Coronavirus 2" OR "NCOV" OR "2019 NCOV" OR "severe acute respiratory syndrome" OR "severe acute respiratory syndrome-related coronavirus") AND NOFT("Neurodevelopment" OR "neurodevelopmental disorders" OR "developmental disabilities" OR "developmental disorder")

The selection of studies was carried out in two phases. In Phase 1, two reviewers (P.A.M. and T.V.A.) examined, independently and blindly, the titles and abstracts of all citations retrieved from the identified electronic databases. Articles that did not meet the inclusion criteria were excluded. In Phase 2, the same reviewers applied the eligibility criteria to the full texts of the articles included in Phase 1 independently. In cases of persistent disagreement after discussion between the reviewers, a third reviewer (A.G.D.S.) was involved in the final decision.

To calibrate the selection of articles by the two reviewers, the Kappa agreement coefficient was calculated through a partial literature search. The Phase 1 reading only began after obtaining a value greater than 0.7 for this index. The Rayyan website (http://rayyan.qcri.org) was used to blind the reading of the retrieved references and ensure independence and confidentiality in both phases. To facilitate this, a team member (M.R.), who did not participate in the selection, acted as a moderator.

Two reviewers (P.A.M. and T.V.A.) collected information from the selected articles, which was later discussed. The extracted data included study characteristics (author, year of publication, country, and study design), population characteristics (sample size, average age, gender, and neurodevelopmental assessment method), and their results. Any discrepancies found were resolved through discussion and mutual agreement between the reviewers. If the two reviewers could not reach a consensus, a third reviewer (A.G.D.S.) intervened to make the final decision.

In cases of missing data or incomplete information in the articles, up to three attempts were made to contact the first and last authors, with intervals of one week between each attempt, to obtain further clarification. If there was no response after these attempts, the article was excluded with the appropriate justification.

Frequency data (absolute or relative) from the neurodevelopmental disorder screening in the sample, along with the respective sample size of the included studies, were extracted. Data were extracted for the exposed group and for the control group when available. Data derived from neurodevelopmental disorder screening using non-validated methods were not accepted, even if their results were available.

The risk of bias was assessed separately by three reviewers (P.A.M., T.V.A., and A.D.S.) using the Joanna Briggs Institute Critical Assessment Checklist^22^ for observational and cohort studies, the included articles were classified as "high risk," "moderate risk," and "low risk" when the domains with "yes" responses represented 0–49%, 50–69%, and 70% or more, respectively. A consensus meeting among the reviewers was held to resolve any disagreements. The web application robvis (https://www.riskofbias.info/welcome/robvis-visualization-tool) was used to generate the figures.

As the outcomes were reported as binary data, the risk ratio between the exposed group and a control group was used as the effect measure. Additionally, the event proportion in the exposed group was calculated when possible. Thus, a random effects meta-analysis was performed using the R programming language in the R Studio development environment (version 1.2.1335, RStudio Inc., Boston, USA) to assess the association between exposure to SARS-CoV-2 during any period of pregnancy and neurodevelopmental delay in infants (0–2 years). The study weights were determined using the Mantel-Haenszel method, and for the variance calculation, expressed by Tau2 values, the DerSimonian-Laird estimator was used. The Freeman-Tukey double arcsine transformation method was applied to ensure the data followed an approximately normal distribution. Confidence intervals of 95% were calculated for each meta-analysis.

Due to the inability to conduct an assessment using a funnel plot (n<10) to minimize the likelihood of publication bias, a comprehensive search was performed across multiple databases, including one in a language other than English (LILACS) and gray literature.

## RESULTS

**Figure 1:**
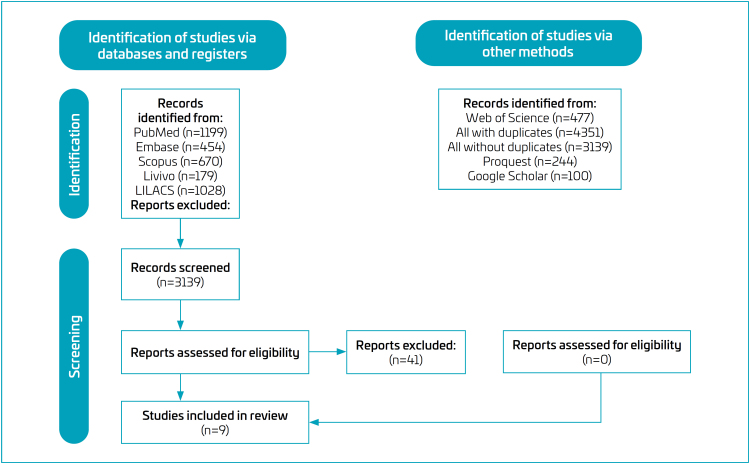
Flowchart of the literature search and selection criteria.^23^

Initially, 3139 articles were identified. After removing duplicates, 46 articles were selected for full-text review in Phase 2, of which 41 were excluded, resulting in five articles included. Of the 41 articles excluded after full-text screening, the most frequent reasons were study type not meeting eligibility criteria (literature reviews, case reports, personal opinions, letters, posters, or conference abstracts; n=15) and use of developmental assessment tools other than Denver II, ASQ-3, or GDS (n=12). In addition, seven articles did not provide an evaluation of the neurodevelopment of children with prenatal exposure to SARS-CoV-2, six corresponded to ongoing studies without available results, and one study focused on children with pre-existing comorbidities or perinatal complications unrelated to COVID-19. A search of the references of these articles, using Citation Chaser, added 319 new articles, of which four were selected after applying the eligibility criteria, bringing the total to nine selected articles (Figure 1)^23^. No articles from gray literature were included.

Articles published in English from Brazil, China, Spain, the USA, Iran, and Kuwait were included. The main outcome assessed was the prevalence of neurodevelopmental disorders in infants exposed to SARS-CoV-2 during pregnancy. The validated screening scales ASQ3 and Bayley III were used for evaluation. The included articles reported the assessment of infants between 3 and 24 months of age, with sample sizes ranging from 28 to 342 neonates (Table 2)^24-32^.

**Table 2:** Characteristics of the included studies (n=9).

Study, Country	Study design	Sample size	Assessment method	Results
Ayede et al.^24^ Kuwait	Cross-sectional	298	ASQ-3	A delay in development was identified in 30 (10.1%) of the babies.
Colom et al.^25^ Spain	Cross-sectional	55	Bayley-III	96% had normal development, 1 had a developmental disorder, and 1 had a mild developmental delay.
Fajardo-Martinez et al.^26^ EUA	Cohort	300 (172 exposed and 128 unexposed)	Bayley-III e ASQ-3	In the assessment using Bayley-III, 12 children (9.4%) from the exposed group showed signs of severe developmental delay, while 2 (1.6%) from the non-exposed group did. Using the ASQ-3, 80 children scored below the threshold.
Mulkey et al.^27^ EUA	Cross-sectional	28	ASQ-3	17 babies showed scores close to or below the threshold considered normal.
Namakin et al.^28^ Irã	Cohort	342 (161 exposed and 181 unexposed)	ASQ-3	This study found no significant differences between babies exposed and unexposed to COVID-19 regarding neurological development. There were no differences in the average scores of developmental tests, except in the communication area.
Santos et al.^29^ Brasil	Cohort	137 (69 exposed and 68 unexposed)	ASQ-3	The prevalence of atypical development was 35.7% at 4 months, 7% at 6 months, and 32.1% at 12 months. No association was found between the ASQ-3 scores and the severity of maternal infection, nor with the trimester of infection.
Shah et al.^30^ EUA	Cohort	51	ASQ-3	Of the 51 individuals, twelve (24%) were below the cutoff point, and twenty-seven (53%) were below or close to the cutoff point in at least one developmental domain.
Shuffrey et al.^31^ EUA	Cohort	255 (114 exposed and 141 unexposed)	ASQ-3	In utero exposure to maternal SARS-CoV-2 infection was not associated with significant differences in any subdomain of the ASQ-3, regardless of the timing or severity of the infection.
Wang et al.^32^ China	Cross-sectional	57	ASQ-3	The overall proportion in the domains: communication, gross motor, fine motor, problem-solving, or personal-social ranged from 13.5 to 23.1%, while in the socioemotional domain, the proportion was 86.4%.

ASQ-3: Ages and Stages Questionnaires – third edition; Bayley-III: Bayley Scales of Infant and Toddler Development – third edition; ^3^SARS-CoV-2: Severe Acute Respiratory Syndrome Coronavirus.

**Figure 2:**
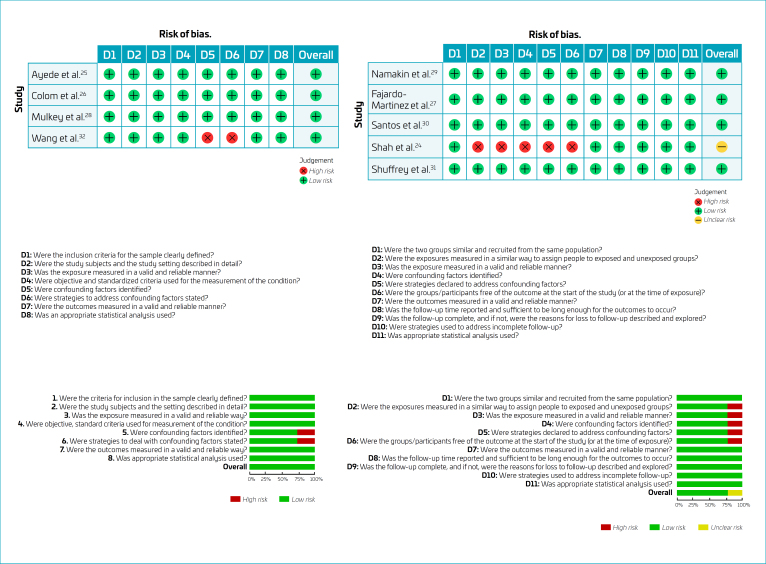
Risk of bias.

Of the nine studies included in this review, only one was classified as having a "moderate risk" of bias,^30^ while the remaining studies were considered to have a "low risk" of bias (24–31). The identified weaknesses focused mainly on the identification and management of confounding factors (Figure 2)^24-32^.

Most of the studies used the ASQ-3 scale as the sole tool to assess the neurodevelopment of the infants.^23,24,27-31^ Only one study, in addition to the ASQ-3, also used the Bayley-III scale.^26^ The ASQ-3 scale is a tool that includes five different domains for evaluation. Two of the included studies presented the results of each domain independently,^28,31^ both studies obtained the lowest scores in the domain related to fine motor skills.

Regarding the gestational period in which the mothers were infected, six articles reported a predominance of infection in the third trimester of pregnancy,^24-26,28,30,32^ while two studies reported a predominance of infection in the second trimester.^29,31^ One article did not describe the period in which the pregnant women were infected.^27^

Three studies compared the impact on the neurodevelopment of infants exposed and unexposed to SARS-CoV-2 during pregnancy.^26,29,31^ Two of them concluded that there was a negative impact on the neurodevelopment of infants exposed to the virus in utero compared to the control groups.^26,29^ The third study revealed that exposure to the virus alone was not a determining factor for delays in neurodevelopment; however, being born during a pandemic period was.^31^

Among the studies that did not compare exposed and unexposed infant groups, three reported significant delays in neurodevelopment. One study associated a higher prevalence of delays in infants exposed to the virus between the first and second trimesters of pregnancy, particularly in premature neonates.^24^ Another study found no other specific associations^27^ and a third concluded that, even in full-term infants, the prevalence of neurodevelopmental disorders is still significant.^30^

On the other hand, two studies revealed that most of the exposed infants did not exhibit delays in neurodevelopment^25^ and that exposure to the virus was not associated with the risk of low birth weight or preterm birth.^28^ In one study, the proportion of infants identified with potential risk of delay ranged from 13.5% to 23.1%, considering the domains assessed by the ASQ-3. However, the authors concluded that a larger sample of infants is needed for more definitive conclusions.^32^

**Figura 3:**
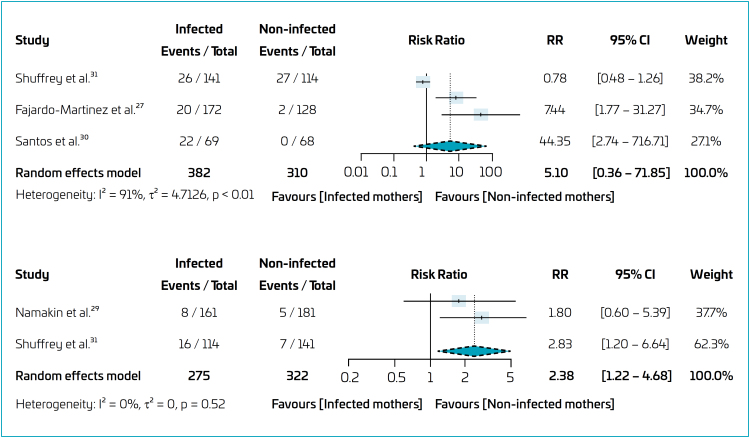
Forest plots

A meta-analysis was conducted to assess the risk of delay in fine motor development and neurodevelopment between the exposed and unexposed groups (Figure 3)^26,28,29,31^. The analysis included three studies,^26,29,31^ for the comparative quantitative synthesis between the exposed and unexposed groups regarding neurodevelopmental disorders. The results did not show a significant difference between the groups [RR=5.10; 95%CI 0.36–71.85; I^2^=91%]. For the "fine motor" domain, two studies were included,^28,31^ and the exposed group showed a 2.38 times higher risk [95%CI 1.22–4.68] compared to the non-exposed group, with low heterogeneity in the analysis (I^2^=0%).

## DISCUSSION

The COVID-19 pandemic, caused by the SARS-CoV-2 virus, placed a significant burden on global healthcare systems, revealing vulnerabilities in public health policies. The high morbidity and mortality rates associated with the virus highlighted the urgency of strengthening management strategies and responding to health crises effectively.^33-35^ Pregnant women, classified as a high-risk group, are more likely to develop complications when infected with SARS-CoV-2.^36,37^ Given the magnitude of the pandemic, understanding the relationship between the infection and the neurodevelopment of infants exposed in utero is crucial for anticipating the long-term consequences on child health.^4,38^

Most of the pregnant women studied contracted SARS-CoV-2 during the third trimester of pregnancy, accounting for 49% of the cases. This result may be attributed to the fact that, in some countries, universal testing is predominantly conducted near the time of delivery.^39^

Comparatively, studies that evaluated infants exposed to the infection and unexposed groups did not show statistically significant differences in neurodevelopment. Additional research highlighted that not only in utero exposure to the virus but also stressors, such as social isolation and financial instability during the pandemic, may impair infant development, being associated with depressive symptoms and anxiety, which are known to affect neurodevelopment.^40^ Some observational studies identified significant delays in neurodevelopment, although without comparative groups, which weakens the robustness of the results. Prematurity was emphasized as an important factor in this context, with pregnant women experiencing severe COVID-19 symptoms being more likely to have preterm births, one of the main factors affecting typical neurological development.^41,42^ In the present study, despite the lack of statistical significance, a large variability in the results was observed, indicated by the high heterogeneity and wide confidence intervals. This suggests uncertainty in the assessment of this outcome, and further studies are needed to strengthen the robustness of these findings.

Considering the ASQ scale, widely used in the studies, infants exposed to SARS-CoV-2 showed a 2.38% increase in the risk of fine motor deficits, corroborated by other systematic reviews.^18,40-42^ Although the overall quantitative analysis did not identify a statistically significant difference in neurodevelopment between infants exposed and unexposed to SARS-CoV-2 during pregnancy, a 2.38-fold increased risk of delay in the fine motor domain was observed among exposed infants. This finding, with low heterogeneity among the included studies (I^2^ = 0%), supports the hypothesis that gestational infection may affect specific aspects of neuromotor development, even in the absence of global impairment. Two studies that individually analyzed the domains of the ASQ-3 scale highlighted fine motor skills as the domain with the highest proportion of below-expected scores, suggesting a particular vulnerability of this aspect of neurodevelopment.^27,29^ Although the magnitude of the risk is modest, the clinical impact may be relevant in a public health context, especially considering that early deficits in fine motor skills can affect visuomotor coordination and future academic performance.

In summary, the results of this systematic review and meta-analysis do not allow for a definitive association between in utero exposure to SARS-CoV-2 and neurodevelopmental disorders in infants. Several methodological limitations were identified among the studies included in this review, which require caution when interpreting the findings. First, most studies employed cross-sectional observational or cohort designs with limited follow-up, hindering causal inference and the assessment of long-term outcomes. Second, there was wide variation in the timing of neurodevelopmental assessments (ranging from 3 to 24 months of age), as well as in the instruments used, with the ASQ-3 predominating — a useful screening tool but less sensitive than more comprehensive scales such as the Bayley-III. Furthermore, many studies did not report essential information, such as gestational age at birth, Apgar scores, or severity of maternal infection, which compromises the analysis of potential confounding factors. Finally, significant heterogeneity among the studies (I^2^>90% in several analyses) indicates variability in methods, populations, and outcome definitions, limiting the generalizability of the results.

The pandemic brought numerous challenges and uncertainties, highlighting the limited understanding of long-term impacts on health and society. In this context, further extensive and detailed studies are needed, with appropriate sampling and robust methodologies, to monitor and more accurately assess infants exposed to the virus during pregnancy, considering the importance of longitudinal follow-up of these children to better understand the potential effects of SARS-CoV-2 on child development.

Despite the association of a higher risk of delays in fine motor skills, the impact on the neurodevelopment of children born to mothers exposed to SARS-CoV-2 during pregnancy remains uncertain. Current evidence highlights the need for new studies with appropriate sampling and robust methodologies to evaluate this outcome more precisely. Furthermore, longitudinal follow-up of these children is crucial, given the still-limited knowledge of the long-term potential impacts that SARS-CoV-2 may have on child development.

## Data Availability

The database that originated the article is available with the corresponding author.
